# The impact of social distancing measures (quarantine) policy on tertiary education and medical consultations in China during the COVID-19 pandemic

**DOI:** 10.3389/fpubh.2024.1365805

**Published:** 2024-03-05

**Authors:** Zhuoer Wang, Shisan Bao

**Affiliations:** ^1^School of Public Health, Lanzhou University, Lanzhou, Gansu, China; ^2^School of Biomedical Engineering, The University of Sydney, Sydney, NSW, Australia

**Keywords:** COVID-19, pandemic, immunity, lockdown, long COVID-19

## Introduction

The 2019 novel coronavirus (COVID-19) pandemic prompted the implementation of rigorous social distancing measures, including quarantine protocols, to curb the virus's spread. This opinion focuses on the consequences of such measures on two critical aspects of society in China: tertiary education and healthcare. By examining the shifts in educational practices and healthcare delivery, the current opinion aims to understand the implications for students and educators in adapting to remote learning modalities, the efficacy of online education platforms, and their academic performance. The current opinion also focused on the management of patients during these unprecedented times. Additionally, the research explores innovative approaches adopted by educational institutions to maintain academic continuity and support the patient's wellbeing from their communities.

## Education sector impact: adapting education during the COVID-19 pandemic

This opinion aims to analyse the challenges faced by students in adapting to remote learning modalities, the efficacy of online education platforms, and the psychological effects on both students and educators. Additionally, the research explores innovative approaches adopted by educational institutions to maintain academic continuity and support the wellbeing of their communities.

The imposition of COVID-19 restrictions, including social distancing and limited mobility, prompted a nationwide lockdown (1, 2), leading to the sudden closure of schools and universities. In response, the Ministry of Education, China, decided to suspend in-person classes but not cancel them entirely, necessitating a shift from traditional teaching to online instruction (3).

Amid the initial and subsequent waves of the COVID-19 outbreak, educational institutions across China transitioned to online teaching, with Tencent Meeting serving as a crucial delivery platform. Chengdu University, for example, successfully adapted its curriculum for 1^st^-year Medical (4) and Nursing students (5) during the initial extensive 6-month lockdown in 2020 (involving rigorous maintenance of social distancing). Course coordinators seamlessly shifted lectures online, offering live-streamed sessions and recorded content for later review.

Surprisingly, there was a discernible improvement in academic scores across various subjects, including Science, Literature, Biochemistry, Anatomy, and Histology. Even courses heavily reliant on hands-on practice, such as Anatomy and Histology, witnessed higher performance achievements among students (4). The curriculum evolved with the integration of digitized whole slide images, effectively transforming in-person instruction into online tutorials (4, 5). The success of this unintentional shift to online teaching, driven by factors that included focused learning time and convenient class review, is likely to influence future educational practices beyond the pandemic.

Supporting the effectiveness of online modules, a tertiary hospital in Shanghai developed modules for Intern/Registrar training in the clinical management of COVID-19 patients (6). This demonstrates the potential for effective online training even in complex clinical scenarios. Overall, these data suggest that the imposition of social distancing had minimal impact on academic performance amongst college students, while strongly minimizing the risk of viral transmission during the pandemic.

However, it is crucial to acknowledge the adverse psychological consequences of the prolonged 3-year of intermittent lockdown, particularly on individuals with pre-existing mental health issues. Approximately 35% of participants reported distress, including feelings of anxiety and depression, with the closure of schools having unfavorable effects, especially on children and adolescents (7). This underscores the importance of addressing mental health concerns alongside the adaptation of novel educational strategies.

### Impact of stringent measures on healthcare access during the COVID-19 pandemic

Social distancing measures, while essential for public health, posed challenges to routine healthcare services. The opinion examines the impact on patient consultations, focusing on changes in medical infrastructure, telemedicine adoption, and the overall patient experience. By assessing the effectiveness of these adaptations, the opinion aims to provide insights into the evolving dynamics of healthcare delivery during the pandemic.

The aftermath of the stringent national lockdown in the initial months of 2020 led to significant restrictions on patients' visits to hospitals and clinics, directing those with fever symptoms to designated Fever clinics (8). Access to medical care required Quick Response code (QR code) scanning and a recent, within 72 h, negative polymerase chain reaction (PCR) test. The most significant concern in relation to this screening system was medical access failure that may occur in the case of an emergency, e.g., asthmatic attack (9) or dialysis patients (10). Additionally, some patients who had attended Emergency were required to wait desperately for medications, but were unable to access the medication until their QR code turned into the green color (11).

Primary care access underwent substantial changes due to pandemic-related administrative processes, resulting in over a 25% reduction in patient visits in the first 6 months of 2020. To curb viral transmission, general practitioner (GP) consultations were moved outdoors, utilizing tents to facilitate fresh air circulation (8). Notably, visits for respiratory issues decreased, likely influenced by measures such as social distancing, mask-wearing, and avoiding gatherings. Patients visiting clinics sought larger prescriptions to minimize frequent visits, while anxiety and depression-related visits increased, possibly linked to the fear of infection and long-term lockdown (8). These data suggest that keep social distancing and/or lockdown provided a useful approach for reducing/minimizing viral transmission among patients and/or the general population.

Subsequently, during the Omicron variant outbreak of severe acute respiratory syndrome coronavirus 2 (SARS-CoV-2) (12), the Shanghai authorities implemented the strictest lockdown measures ever, i.e., nobody was allowed to leave their homes under any circumstances for an entire 2 months (13). As a direct consequence of this lockdown, face-to-face medical consultations were immediately suspended (13). In response to this formidable challenge, GPs were determined to provide seamless care for patients with chronic diseases. For those individuals with comprehensive medical records stored in the hospital's electronic file system (8), GPs initiated telephone consultations to assess their current health status. Urgent cases were directed to designated COVID-19 Hospitals for emergency care. Surprisingly, there was no significant correlation between patient visits and COVID-19 infection rates, indicating the effectiveness of stringent control measures, i.e., maintaining social distance. However, these rigorous measures overlooked the adverse mental health impacts.

The debate over the necessity of extensive restrictions and prolonged lockdowns continues, with a need to weigh the benefits against potential adverse impacts on the wellbeing of Shanghai residents.

### Abolition of zero tolerance policy

In the light of the rapid and unstoppable spread of the Omicron variant throughout China, a significant shift in the preventative landscape occurred on December 7, 2022, when Chinese authorities made a noteworthy overnight decision to abolish abruptly the dynamic-zero policy that had been in place for 3 years. This pivotal decision was accompanied by the re-classification of the COVID-19 virus as a category-B infectious pathogen (14), which corresponds to a classification as moderately easy to disseminate; resulting in moderate morbidity and low mortality rates. Furthermore, individuals were actively encouraged to resume normal social activities with the goal of reaching a turning point in the overall level of infections within the community, with the ultimate aim of rapidly reducing the number of acute infectious cases circulating in the community. However, to achieve this objective, essentially the authorities aimed to introduce herd immunity *via* by initially causing rapid and widespread acute infections throughout the community (15).

However, there is a potential issue raised from such an abrupted action. Specifically, what are the consequences of the lack of development of host immunity against SARS-CoV_2_ variants during the 3 years of lockdown? Despite the claim that the vaccination rate had reached >90%, in Shanghai patients' visits to hospital were noted to surge dramatically at the time of the abolition of the COVID-19 zero-tolerance policy, raising lessons for any future response (15). Such findings suggest that people may be better off if they had been offered more effective targeted vaccination, in addition to progressive exposure over time to small amounts SARS-CoV_2_ virus, to progressively build some host immunity within the community, *via* the gradual easing of restrictions, e.g. by allowing community activity (shopping, working etc.) while maintaining a suitable level of social distancing and mask use. The dramatic cessation of zero tolerance, incorporating the complete abolition of social distancing policy is arguably not the best approach in dealing with such a challenge.

### Ongoing viral mutation and long COVID-19—Maintaining social distancing and mask usage

While WHO has declared an end to the COVID-19 pandemic, the repercussions of COVID-19 persist due to new mutation(s). Subsequently, the mutated virus has been reintroduced into the community. Even though there is no longer a pandemic, it raises the question of whether continued monitoring of this relatively minor virus is necessary for the wellbeing of the older adult and immunocompromised population in China, as has been suggested in Australia (16).

Furthermore, cases of long COVID-19 persist both in China (17) and internationally (18, 19). In addition to immediate symptoms, a significant number of patients experience post-COVID-19 syndrome, commonly known as long COVID (20). Long COVID-19 affects the respiratory (21), neurological (22), cardiovascular (23), muscular (24), and digestive systems, with many documented cases within China. This necessitates substantial and costly therapeutic interventions. Approximately 10% of patients are estimated to experience persistent organ damage following infection with the SARS-CoV-2 virus. The precise underlying mechanism of long COVID-19 is still under investigation, as are the diagnostic and treatment options for this condition. The linkage between social distancing and long-COVID-19 remains to be explored.

Although the use of social distancing and facial masks is no longer a mandatory requirement for Chinese residents, there are still quite a number of people voluntarily wearing facial masks and minimizing their exposure to large gatherings, considering recent cluster outbreaks of respiratory issues over the last few months (25).

## Conclusion

The findings of this study contribute to a comprehensive understanding of how social distancing measures, particularly quarantine, have influenced education outcomes and medical consultations in China during the COVID-19 pandemic. By highlighting challenges and innovative solutions, the research provides future preparedness strategies for similar global health crises, emphasizing the importance of flexibility and technology integration in sustaining essential societal functions. Finally, this opinion prompts consideration of the challenges and strategies in education and healthcare against similar global health crises. Additionally, the psychological effects of prolonged and restricted quarantine on students, educators, and patients should be taken into account in preparation for unforeseen future circumstances.

## Author contributions

ZW: Writing—original draft. SB: Conceptualization, Writing—review & editing.
